# Neuromuscular blocking agents in patients with acute respiratory distress syndrome: a summary of the current evidence from three randomized controlled trials

**DOI:** 10.1186/2110-5820-2-33

**Published:** 2012-07-26

**Authors:** Ary Serpa Neto, Victor Galvão Moura Pereira, Daniel Crepaldi Espósito, Maria Cecília Toledo Damasceno, Marcus J Schultz

**Affiliations:** 1 Medical Intensive Care Unit, ABC Medical School (FMABC), Av. Lauro Gomes, Santo André, 2000, Brazil; 2 Department of Intensive Care Medicine & Laboratory of Experimental Intensive Care and Anesthesiology; Academic Medical Center, University of Amsterdam, Amsterdam, The Netherlands

**Keywords:** ARDS, Neuromuscular blocking agents, Meta-analysis, Review

## Abstract

**Background:**

Acute respiratory distress syndrome (ARDS) is a potentially fatal disease with high mortality. Our aim was to summarize the current evidence for use of neuromuscular blocking agents (NMBA) in the early phase of ARDS.

**Methods:**

Systematic review and meta-analysis of publications between 1966 and 2012. The Medline and CENTRAL databases were searched for studies on NMBA in patients with ARDS. The meta-analysis was limited to: 1) randomized controlled trials; 02) adult human patients with ARDS or acute lung injury; and 03) use of any NMBA in one arm of the study compared with another arm without NMBA. The outcomes assessed were: overall mortality, ventilator-free days, time of mechanical ventilation, adverse events, changes in gas exchange, in ventilator settings, and in respiratory mechanics.

**Results:**

Three randomized controlled trials covering 431 participants were included. Patients treated with NMBA showed less mortality (Risk ratio, 0.71 [95 % CI, 0.55 – 0.90]; number needed to treat, 1 – 7), more ventilator free days at day 28 (*p* = 0.020), higher PaO_2_ to FiO_2_ ratios (*p* = 0.004), and less barotraumas (*p* = 0.030). The incidence of critical illness neuromyopathy was similar (*p* = 0.540).

**Conclusions:**

The use of NMBA in the early phase of ARDS improves outcome.

## Background

Acute respiratory distress syndrome (ARDS) is a potentially fatal disease with high mortality even with the use of protective ventilation strategy [1]. Several therapies were tested in patients with ARDS like prone positioning, corticosteroids, inhalation of nitric oxide, high frequency oscillatory ventilation, recruitment maneuvers and, in the most severe cases, extracorporeal membrane oxygenation (ECMO) [2].

Recently, Papazian *et al*[3] showed that the use of a neuromuscular blocking agent (NMBA) early in the course of ARDS improves the overall survival and increases the time off the ventilator. This study has some limitations as: 01) patients who are not paralyzed can trigger the ventilator and paralyzed patients cannot so, adequate blinding would seen to have been nearly impossible; 02) the neuromuscular blockade was not assessed by the train-of-fours; and 03) the assessment of muscle weakness had some limitations. Current guidelines indicate that NMBA are appropriate for facilitating mechanical ventilation when sedation alone is inadequate, most notably in patients with severe gas-exchange impairments, like in patients with ARDS [4].

The potential use of NMBA in patients with ARDS was tested in a few randomized controlled trials. In front of the limitations of the larger study in this field we conducted a systematic review and meta-analysis of the literature to summarize briefly the current evidence for the use of NMBA in patients with ARDS.

## Methods

### Literature search and data extraction

The online database of MedLine (1966 – 2012) and Cochrane Register of Controlled Trials were searched for studies that fulfill the following inclusion criteria: 1) randomized controlled trials; 02) adult human patients (age > 18 years) with ARDS or acute lung injury (AECC criteria in the first 48 hours); [5] and 03) use of any NMBA in one arm of the study compared with another arm without NMBA. There is no language restriction.

The following terms were combined in the search strategy: (acute respiratory distress syndrome [MeSH] OR acute lung injury [MeSH]) AND (neuromuscular blocking agents [MeSH] OR neuromuscular blockade [MeSH] OR vecuronium bromid [MeSH] OR atracurium [MeSH] OR cisatracurium [MeSH] OR pancuronium [MeSH] OR rocuronium [MeSH]). When we found duplicate reports of the same study in preliminary abstracts and articles, we analyzed data from the most complete data set.

Data were independently extracted from each report by three authors, using a data recording form developed for this purpose. After extraction, data were reviewed and compared by the first author. Instances of disagreement between the two other extractors were solved by a consensus among the investigators. The quality of each study was assessed as suggested by the Grading of Recommendation Assessment Development and Evaluation Working Group (GRADE). Also, we used the GRADE approach to summarize the quality of evidence for each outcome [6].

### Outcomes and data analysis

The primary outcome was overall mortality in patients treated with NMBA vs. patients not treated with NMBA. The secondary outcomes included: ventilator free days, duration of mechanical ventilation, adverse events (barotrauma and neuromyopathy), changes in gas exchange (PaCO_2_, PaO_2_ / FiO_2_), ventilatory settings (PEEP, FiO_2_, tidal volume), and respiratory mechanics (plateau pressure).

We extracted data regarding the study design, patient characteristics, overall survival, time to extubation, incidence of adverse events, and mean change in arterial blood gases. For the analysis of survival, we calculated a pooled estimate of risk ratio (RR) in the individual studies using a fixed effect model according to Mantel and Haenszel and graphically represented these results using forest plot graphs. For continuous variables, we used the standardized mean difference (SMD) which is the difference in means divided by a standard deviation. The homogeneity assumption was checked by a χ [2] test with a *df* equal to the number of analyzed studies minus 1.

All analyses were conducted with Review Manager v.5.1.1 and SPSS v.16.0.1. The Summary of Findings (SoF) tables were created in GRADEpro 3.6. For all analyses p values < .05 were considered significant.

## Results

**Figure 1:**
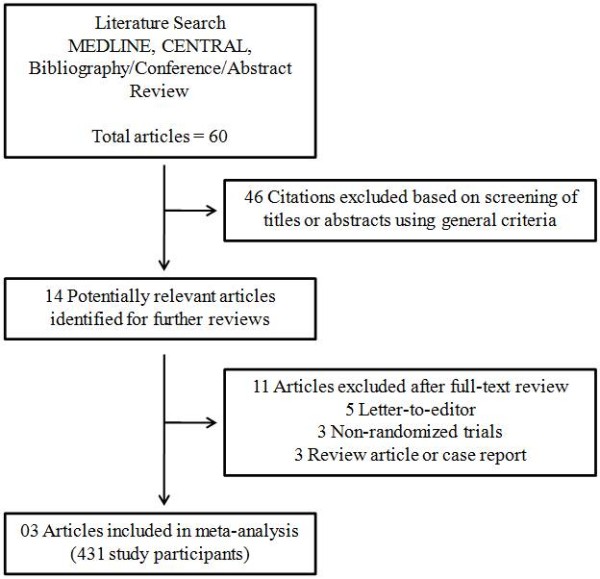
Literature search strategy.

The comprehensive literature search yielded 60 references, of which 46 articles were excluded during the first screening, which was based on abstracts or titles, leaving 14 articles for full text review. During this review, 11 articles were excluded for the following reasons: letter to editor (n = 5); non-randomized trial (n = 3); review article (n = 2); and case report (n = 1). Finally, three articles (431 participants) were included in the final analysis [3,7,8] (Figure 1).

All three studies analyzed were randomized controlled trials and uses placebo in the control arm. All three studies used cisatracurium during 48 hours for myorelaxation, and two studies assessed neuromuscular blockade with the train-of-four [6,7]. The mean follow-up for the assessment of gas exchange was 136.00 ± 27.71 hours. Characteristics of the studies analyzed are show in Table 1 and the study quality assessment is show in Table 2.

**Table 1:** Characteristics of the studies included

**Characteristics**	**Gainnier M, 2004**^**7**^		**Forel JM, 2006**^**8**^		**Papazian L, 2010**^**3**^	
	**NMBA**	**Control**	**NMBA**	**Control**	**NMBA**	**Control**
Number of centers	Four		Three		Twenty	
Number of participants	28	28	18	18	177	162
Age, years	59.8 ± 17.5	61.5 ± 14.6	52.0 ± 16.0	61.0 ± 18.0	58.0 ± 16.0	58 ± 15.0
Lung injury score	2.89 ± 0.40	2.93 ± 0.42	3.0 ± 0.2	2.8 ± 0.4	---	---
SAPS II	41.8 ± 10.4	45.4 ± 10.5	47.0 ± 15.0	49.0 ± 19.0	50.0 ± 16.0	47.0 ± 14.0
Drug used	Cistracurium	Placebo	Cistracurium	Placebo	Cisatracurium	Placebo
Sedation strategy	Ramsay 6^*^	Ramsay 6^*^	Ramsay 6^*^	Ramsay 6^*^	Ramsay 6^*^	Ramsay 6^*^
Assessment of blockade	TOF	None	TOF	None	None	None
Maximal time to randomization, hours	36	36	48	48	48	48
Time with NMBA, hours	48		48		48	
Onset of ARDS, days	0.96 ± 0.79	1.14 ± 1.72	1.0 ± 0.8	1.2 ± 0.8	0.75 ± 0.41	0.62 ± 0.32
Days receiving mechanical ventilation	2.7 ± 2.6	3.4 ± 3.5	---	---	0.91 ± 0.87	0.87 ± 0.74
Baseline PaO_2_ / FiO_2_	130 ± 34	119 ± 31	105 ± 22	125 ± 20	106.0 ± 36.0	115.0 ± 41.0^**^
Baseline PaCO_2_, mmHg	48.3 ± 9.0	47.4 ± 11.2	51.1 ± 9.9	47.2 ± 9.8	47.0 ± 11.0	47.0 ± 11.0
Baseline PEEP, cmH_2_O	11.1 ± 2.8	10.9 ± 2.4	13.2 ± 2.7	11.0 ± 2.7	9.2 ± 3.2	9.2 ± 3.5
Baseline V_T_, mL/kg	7.1 ± 1.1	7.4 ± 1.9	6.5 ± 0.7	7.0 ± 0.7	6.55 ± 1.12	6.48 ± 0.92
Baseline FiO_2_, %	70.2 ± 17.0	67.3 ± 15.8	80.0 ± 15.0	71.0 ± 19.0	79.0 ± 19.0	77.0 ± 22.0
Baseline Plateau pressure, cmH_2_O	27.1 ± 6.2	26.1 ± 4.0	27.5 ± 4.4	24.8 ± 5.7	25.0 ± 5.1	24.4 ± 4.7
Time of assessment of oxygenation, hours	120	120	120	120	168	168
Duration of mechanical ventilation, days	20.9 ± 15.0	21.2 ± 17.4	20.0 ± 11.6	18.0 ± 8.3	---	---
Ventilator-free days at day 28, days	3.7 ± 7.2	1.7 ± 5.3	6.0 ± 8.6	5.4 ± 6.4	10.6 ± 9.7	8.5 ± 9.4^**^
Barotrauma, *n* (%)	0 (0.0)	1 (3.5)	1 (0.0)	1 (0.0)	9 (5.0)	19 (11.7)^**^
Critical illness neuromyopathy, *n* (%)	0 (0.0)	0 (0.0)	1 (5.5)	1 (5.5)	40 (35.7)	28 (36.3)
ICU mortality, *n* (%)	13 (46.4)	20 (71.4)	5 (27.8)	10 (55.6)	52 (29.3)	63 (38.8)
Mortality at day 28 after inclusion, *n* (%)	10 (37.5)	17 (60.7)	---	---	42 (23.7)	54 (33.3)^**^

NMBA: Neuromuscular blocking agents; SAPS: Simplified acute physiology score; ARDS: Acute respiratory distress syndrome; PEEP: Positive end expiratory pressure; V_T_: Tidal volume; FiO_2_: Fraction of inspired oxygen; ICU: Intensive care unit; TOF: train-of-fours.

*: Midazolam + Sufentanil.

**: *p* ≤ 0.05.

**Table 2:** Assessment of study quality

**Studies**	**Allocation Concealment**	**Blinding**	**ITT Analysis**	**Lost to Follow-up**	**Early Stopping**	**Baseline similarity**
Gainnier M, 2004	Yes (Not specified)	Yes	Yes	No	No	Yes
Forel JM, 2006	Yes (Not specified)	Yes	No	No	No	Yes
Papazian L, 2010	Yes (Computer generated)	Yes	Yes	No	No	Yes

ITT: Intention-to-treat.

**Figure 2:**
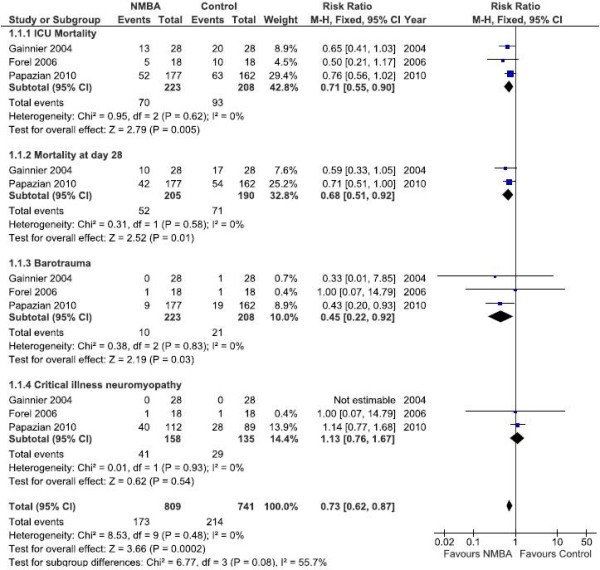
Effect of neuromuscular blockade in patients with ARDS at the end of the follow-up period for each study.

Seventy out of 223 patients (31.4 %) assigned to neuromuscular blockade and 93 out of 208 patients (44.7 %) assigned as controls died during ICU stay (Risk ratio [RR], 0.71 [95 % CI, 0.55 – 0.90]; number needed to treat [NNT], 1 – 7). After analyzed 385 patients from two studies [3,7], this finding was similar after 28 days of follow-up (RR, 0.69 [95 % CI, 0.51 – 0.92]; NNT, 1 – 8). Patients treated with NMBA needed less days of mechanical ventilation with a higher number of ventilator-free days at day 28 (*p* = 0.0020). At the final of follow-up, patients treated with NMBA showed an increase in PaO_2_ to FiO_2_ ratio (*p* = 0.004), at lower FiO_2_ (*p* = 0.002). The incidence of barotrauma was lower in patients under neuromuscular blockade (*p* = 0.030) while the incidence of critical illness neuromyopathy was similar in both groups (*p* = 0.540). (Table 3 and Figure 2).

**Table 3:** Baseline characteristics and outcomes of the patients

	**Cisatracurium**	**Control**	***p*value**	**SMD / RR**	**Heterogeneity**	***p value***
	**(*n*= 223)**	**(*n*= 208)**		**(95% Confidence Interval)**		
Age, years	56.60 ± 4.08	60.16 ± 1.89	0.300^**^			
Lung injury score	2.94 ± 0.07	2.86 ± 0.09	0.667^**^			
SAPS II	46.26 ± 4.14	47.13 ± 1.80	0.999^**^			
Onset of ARDS, days	0.90 ± 0.13	0.98 ± 0.31	0.700^**^			
Days receiving mechanical ventilation	1.80 ± 1.26	2.13 ± 1.78	0.667^**^			
Change in PaO_2_ / FiO_2_^*^	93.66 ± 40.12	42.66 ± 4.04	0.050^**^	SMD: 0.29 (0.09 – 0.49)^¶^	0.320	0.004
Change in PaCO_2_^*^, mmHg	8.76 ± 27.1	10.66 ± 23.80	0.150^**^	SMD: 0.02 (-0.17 – 0.21) ^¶^	0.520	0.800
Change in PEEP^*^, cmH_2_O	- 2.20 ± 1.90	- 0.46 ± 0.56	0.050^**^	SMD: -0.18 (-0.37 – 0.01) ^¶^	0.430	0.060
Change in V_T_^*^, mL/kg	0.28 ± 0.33	0.30 ± 0.11	0.150^**^	SMD: -0.02 (-0.21 – 0.17) ^¶^	0.300	0.850
Change in FiO_2_^*^, %	- 25.40 ± 7.91	- 13.10 ± 7.39	0.050^**^	SMD: -0.31 (-0.50 – -0.12) ^¶^	0.260	0.002
Change in Plateau Pressure^*^, cmH_2_O	- 3.66 ± 1.49	- 0.70 ± 0.60	0.050^**^	SMD: -0.11 (-0.30 – 0.08) ^¶^	0.040	0.250
Ventilator-free days at day 28, days	6.76 ± 3.51	5.20 ± 3.40		SMD: 0.22 (0.03 – 0.41)	0.860	0.020
Barotrauma, *n* (%)	9 (4.0)	20 (9.6)		RR: 0.45 (0.22 – 0.92)	0.830	0.030
Critical illness neuromyopathy, *n* (%)	41 (18.3)	29 (13.9)		RR: 1.13 (0.76 – 1.66)	0.990	0.550
ICU mortality, *n* (%)	70 (31.4)	93 (44.7)		RR: 0.71 (0.55 – 0.90)	0.620	0.005
Mortality at day 28 after inclusion, *n* (%)	52 (23.3)	71 (34.1)		RR: 0.68 (0.51 – 0.92)	0.580	0.010

SAPS: Simplified acute physiology score; ARDS: Acute respiratory distress syndrome; PEEP: Positive end expiratory pressure; V_T_: Tidal volume; FiO_2_: Fraction of inspired oxygen; ICU: Intensive care unit.

^*^ Change in these variables were calculated as follow: (last value of the follow-up) – (baseline value).

^**^ Mann-Whitney Exact Test.

^¶^ SMD calculated with the values in the final period of follow-up.

**Figure 3:**
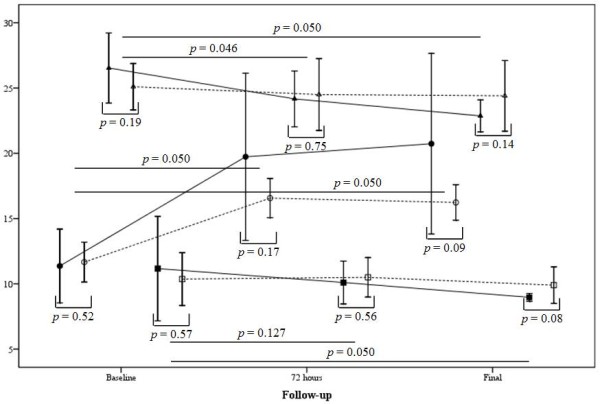
** Changes in PaO**_**2**_**/ FiO**_**2**_**( *circles *), plateau pressure ( *triangles *) and PEEP (*squares *) between patients under NMBA ( *continuous line and closed symbols *) and controls ( *dashed lines and open symbols *).**

PaCO_2_ levels, tidal volume, and plateau pressure were similar in both groups at the final follow-up (*p* = 0.800, *p* = 0.850, and *p* = 0.250, respectively). There was a trend to the use of lower PEEP levels in patients under NMBA (*p* = 0.060), and even with the same tidal volume, patients treated with NMBA had lower plateau pressure at final follow-up, although this not reached significance (*p* = 0.250). However, when we assessed the changes of the variables during the follow-up (last value of the follow-up – baseline values) we founded a higher decrease in PEEP levels (− 2.20 ± 1.90 vs. - 0.46 ± 0.56, for NMBA and controls, respectively; *p* = 0.050), and in plateau pressure (− 3.66 ± 1.49 vs. - 0.70 ± 0.60, for NMBA and controls, respectively; *p* = 0.050). (Table 2). One day after the final of infusion (72 hours) patients in the NMBA group presented a higher increase in PaO_2_ / FiO_2_ ratio and a higher decrease in the plateau pressure (Table 4 and Figure 3).

**Table 4:** Difference of physiological variables one day after the final of the NMBA infusion (72 hours)

	**T0**		**T72**		**Change in Cisatracurium**	**Change in Control**	*p*value^*^
	**Cisatracurium**	**Control**	**Cisatracurium**	**Control**			
	**(*n*= 223)**	**(*n*= 208)**	**(*n*= 223)**	**(*n*= 208)**			
PaO_2_ / FiO_2_	113.6 ± 14.1	119.6 ± 5.03	197.3 ± 32.0	165.6 ± 7.50	83.66 ± 35.92	46.00 ± 4.58	0.050
PaCO_2_, mmHg	48.80 ± 2.09	47.20 ± 0.20	45.43 ± 1.35	44.76 ± 1.59	- 3.36 ± 1.97	- 2.43 ± 1.43	0.658
PEEP, cmH_2_O	11.16 ± 2.00	10.36 ± 1.01	10.10 ± 0.81	10.50 ± 0.75	- 1.06 ± 1.22	0.13 ± 0.47	0.127
V_T_, mL/kg	6.71 ± 0.33	6.96 ± 0.46	6.80 ± 0.34	7.00 ± 0.45	0.08 ± 0.02	0.04 ± 0.05	0.246
FiO_2_, %	76.40 ± 5.39	71.76 ± 4.89	56.00 ± 1.00	59.00 ± 0.00	- 20.40 ± 5.57	- 12.76 ± 4.8	0.127

Positive end expiratory pressure; V_T_: Tidal volume; FiO_2_: Fraction of inspired oxygen; ICU: Intensive care unit.

^*:^ Mann-Whitney Exact Test.

In Table 5, the GRADE evidence profile is provided. This profile evaluates the impact of NMBA in patients with ARDS.

**Table 5:** GRADE evidence profile for impact of NMBA in acute ARDS from systematic review and meta-analysis of randomized controlled trials

**No of studies (No of participants)**		**Quality assessment**					**Summary of findings**		
		**Study limitations**	**Consistency**	**Directness**	**Precision**	**Publication bias**	**Relative effect (95% CI)**	**Best estimate of intervention group**	**Quality**
				**NMBA vs. Control**					
ICU Mortality:									
3 (431)	Moderate limitations^*^	No important inconsistency	Direct	Not important imprecision	Unlikely		0.71 (0.55 – 0.90)	27%	+++, moderate
Mortality at Day 28:									
2 (395)	Moderate limitations^*^	No important inconsistency	Direct	Not important imprecision	Unlikely		0.68 (0.51 – 0.92)	23.7%	+++, moderate
Barotrauma:									
3 (431)	Moderate limitations^*^	No important inconsistency	Direct	Not important imprecision	Unlikely		0.45 (0.22 – 0.92)	0%	+++, moderate
Critical illness neuromyopathy:									
3 (293)	Moderate limitations^*^	No important inconsistency	Direct	Not important imprecision	Unlikely		1.13 (0.76 – 1.67)	0%	+++, moderate

*Unclear allocation concealment in two studies, not analyzed using intention-to-treat in one studies.

## Discussion

We founded evidence that the use of NMBA in the early phase of ARDS improves outcome, demonstrated by a higher overall ICU survival and survival at day 28, a higher number of ventilator-free days at day 28, an increase in PaO_2_ to FiO_2_ ratio, and reduced incidence of barotrauma when compared with the conventional care. Notably, the incidence of critical illness neuromyopathy was similar between patients treated with NMBA and in controls.

Neuromuscular blocking agents block neuromuscular transmission at the neuromuscular junction, causing paralysis of the affected skeletal muscles. The mechanism underlying the beneficial effect of NMBA in patients with ARDS remains unclear. Some authors suggest that NMBA could decrease lung and systemic inflammation [8]. Also, a brief period of paralysis may facilitate lung protective mechanical ventilation by improving patient–ventilator synchrony and allowing for the accurate adjustment of tidal volume and pressure levels, thereby limiting the risk of both asynchrony-related alveolar collapse and regional alveolar pressure increases with overdistention [3]. We showed in our meta-analysis that patients treated with NMBA were ventilated with similar tidal volumes of patients not treated with NMBA, however, the plateau pressure in patients under NMBA showed a higher decrease over time. Finally, it may be necessary to paralyze patients to tolerate hypercapnic acidosis when a lower tidal volume is applied [9].

The use of NMBA requires a deep sedation, and is reasonable to think that patient under NMBA uses more sedation than controls [3]. The only study that assessed this topic showed no difference of duration of sedation with sufentanil and midazolam in patients treated with NMBA and controls [7]. Instead, control patients used sedation for a median of 13 days vs. 6 days in patients treated with NMBA, and this may have happened because the improvement in oxygenation observed in patients treated with NMBA may have allowed an earlier reduction of sedation.

The main safety concern with the use of a NMBA is muscle weakness since several studies suggest that the long-term administration of NMBAs may be associated with the development of neuromuscular weakness in critically ill patients. Cisatracurium appears to be the safer compound, once the occurrence of myopathy is less common than with amino-steroid compounds [10]. We found similar incidence of critical illness neuromyopathy between the groups however, no study performed electromyography exam in the patients. Indeed, the duration of mechanical ventilation was not increased in the NMBA group, and the short duration of use of the NMBA probably explains this finding.

These reported findings should be viewed within the context of the limitations of this study and the research in the field. The main limitation is that all studies analyzed were conducted by the same group of researchers which can make the results biased. However, the largest study was a multicenter trial that included 20 distinct ICUs, reducing the chance of biases. In addition, another limitation of our meta-analysis is that it is dominated by the largest randomized controlled trial by Papazian *et al*[3]. Another important concern is that the largest trial analyzed did not assessed the effectiveness of the neuromuscular blockade with train-of-four stimulation, so we cannot be sure that patients were adequately blocked [11]. Finally, the Medical Research Council scale method used to evaluate muscle weakness was limited to 28 days (or to discharge from the ICU), a duration that may be too brief to recognize muscle weakness in patients who require prolonged mechanical ventilation, particularly if they are slow to awaken [12].

## Conclusion

In conclusion, our meta-analysis demonstrated benefit of use of NMBA early in the course of ARDS without increasing the incidence of critical illness neuromyopathy. These findings needs to be assessed with caution since the studies evaluated came from the same group.

## Competing interests

The authors declare that they have no competing interests.

## Author’s contributions

ASN participated in the concept and design of the study, data acquisition, statistical analysis and interpretation, drafted the manuscript, and revised the manuscript for important intellectual content.

VGMP participated in the data acquisition, and interpretation, drafted the manuscript, and revised the manuscript for important intellectual content.

DCE participated in the concept and design of the study, data acquisition, and interpretation, drafted the manuscript, and revised the manuscript for important intellectual content.

MCTD participated in the data interpretation, drafted the manuscript, and revised the manuscript for important intellectual content.

MJS participated in the data interpretation, drafted the manuscript, and revised the manuscript for important intellectual content. All authors read and approved the final manuscript.
